# Management of Coronavirus Disease 2019 Patients With Lung Cancer: Experience From a Thoracic Oncology Center

**DOI:** 10.3389/fmolb.2021.639676

**Published:** 2021-07-22

**Authors:** David Barros Coelho, Vanessa Santos, David Araújo, Hélder Novais Bastos, Adriana Magalhães, Venceslau Hespanhol, Henrique Queiroga, Natália Cruz-Martins, Maria Gabriela O. Fernandes

**Affiliations:** ^1^Pulmonology Department, Centro Hospitalar Universitário São João, Porto, Portugal; ^2^Faculdade de Medicina, University of Porto, Porto, Portugal; ^3^Institute for Research and Innovation in Health (i3S), University of Porto, Porto, Portugal; ^4^Laboratory of Neuropsychophysiology, Faculty of Psychology and Education Sciences, University of Porto, Porto, Portugal

**Keywords:** COVID-19, lung cancer, infection, thoracic oncology, clinical management

## Abstract

**Background:**

Cancer patients appear to be at a higher risk of complications from coronavirus disease 2019 (COVID-19). Specific data related to lung cancer (LC) patient management, active treatment, and/or recent diagnosis are still very limited. Here, we aimed to investigate the clinical presentation, baseline features, and clinical outcomes of LC patients with COVID-19.

**Methods:**

A retrospective case study was performed at Centro Hospitalar Universitário de São Joao, a tertiary hospital in the North of Portugal. Data from LC patients diagnosed with COVID-19 were collected during the first 10 months of the COVID-19 pandemic (March 2020–January 2021).

**Results:**

Twenty-eight patients with active LC were diagnosed with COVID-19, being adenocarcinoma the most common histological type present (*n* = 13, 46.4%). Sixteen patients had metastatic stage IV LC (61.5%). Twenty-five patients (89.3%) had relevant comorbidities including hypertension (39.3%) and chronic obstructive pulmonary disease (32.1%). For patients undergoing antineoplastic treatment, the median time from the last chemotherapy administration to COVID-19 diagnosis was of 16 days (interquartile range = 13–41 days). Half of patients were previously on corticosteroid therapy. Twenty patients (71.4%) needed hospitalization, 18 received oxygen therapy (64.3%), 3 (10.7%) of them received high-flow nasal cannula with good tolerability, and 1 (3.6%) needed non-invasive ventilation. Hydroxychloroquine and antibiotics were given to 4 (14.3%) and 12 (42.9%) patients, respectively. Seven patients (25%) died at a median time of 5 days following COVID-19 diagnosis.

**Conclusion:**

This is one of the first studies reporting the adverse outcomes associated with COVID-19 in LC patients at same time that adds evidence regarding the need to create protocols and guidelines to reduce the infection risk in such patients.

## Introduction

Coronavirus disease 2019 (COVID-19) is a viral infection triggered by severe acute respiratory syndrome coronavirus 2 (SARS-CoV-2), with high pulmonary impairment. Although the first cases were reported in Hubei, China, in December 2019, the disease spread rapidly around the world and was recognized as a global pandemic by the World Health Organization (WHO) (Guan et al., 2020; Johns Hopkins University, 2020). Among the main patients with features that confer a higher risk of a serious disease state and a higher mortality rate resulting from infection are the elderly and those with underlying comorbidities (Guan et al., 2020; Johns Hopkins University, 2020).

Regarding oncology, data on clinical outcomes in cancer patients are still scarce, although published case reports indicate that cancer leads to worse outcomes. For example, a study conducted in China found that cancer patients were at higher risk of COVID-19 with a worse prognosis than those without cancer (de Marinis et al., 2020; Liang et al., 2020; Passaro et al., 2020; Yu et al., 2020). In addition, the first report from a global database (TERAVOLT) revealed a 33% mortality rate and a low proportion of patients in intensive care or mechanical ventilation (Garassino et al., 2020), an aspect that has already been highlighted by smaller studies (de Marinis et al., 2020; Liang et al., 2020; Passaro et al., 2020; Yu et al., 2020). In this sense, it is important to understand the clinical context and epidemiological features of cancer patients in order to create or even improve the existing protocols in cancer centers to deal with lung cancer (LC) patients in the COVID-19 era. In addition, and given the scarcity of specific data on LC patient management in active treatment and/or with recent diagnosis, it is also of utmost importance to better understand the global scope to develop clinical guidelines in this regard. Anyway, considering the disease burden, age, comorbidities, smoking, and even the immunosuppression state to which such patients are exposed, we hypothesize that they face a higher risk of COVID-19–related complications and even death. Thus, the present study aims to analyze the incidence of cases and the impact of infection control measures in LC patients at a thoracic oncology center.

## Materials and Methods

A retrospective case study was carried out at the Centro Hospitalar Universitário de São João (CHUSJ), a tertiary hospital in the Oporto region, located at the Northern Portugal. In fact, the Oporto region was the epicenter at the time of the first COVID-19 outbreak in Portugal, as it was in the second wave. The first national cases were reported in this region and, according to data from Portuguese public health authorities, six of the 10 most affected municipalities were in the Porto region during the first 2 months of the pandemic.

The Department of Thoracic Oncology is part of the CHUSJ Oncology Unit. Briefly, it is located in an adjacent building to CHUSJ, where consultations, examinations, and radiotherapy and chemotherapy are performed. In this study, data were collected from LC patients with COVID-19 diagnosis in Portugal from March 2020 to January 2021. Active cancer was defined as LC diagnosed or treated in the previous 6 months or recurrent/metastatic cancer.

During this period, a contingency plan was developed by the CHUSJ and the Department of Thoracic Oncology, aiming to (1) reduce the in-person consultation, establishing telemedicine for non-essential care; (2) COVID-19 screening in patients starting treatment (chemotherapy or radiotherapy); (3) regular and adequate cleaning of surfaces and objects in the Oncology Daycare Unit; and (4) patient engagement in social distancing and proper hand cleaning techniques.

Demographic and clinical features, laboratory findings, and chest computed tomography (CT) or chest X-ray images were collected and analyzed.

Coronavirus disease 2019 diagnosis was based on the criteria published by the WHO and confirmed by reverse transcriptase–polymerase chain reaction assay of nasal and/or pharyngeal specimens. Patients with confirmed COVID-19 diagnosis were treated according to the emergency room policy, and ward treatment and follow-up were done by a dedicated team led by the Infectious Diseases Unit.

The study protocol was approved by the ethics committee of CHUSJ.

## Results

A total of 28 patients with active LC were retrospectively identified and analyzed. Baseline demographic and clinical characteristics of patients are summarized in Table 1. Most patients were male (*n* = 20, 71.4%), with median age of 68 years [interquartile range (IQR) = 57–76 years]. All cases presented between March 21, 2020, and January 4, 2021.

**TABLE 1:** Characterization of lung cancer (LC) patients with coronavirus disease 2019 (COVID-19) diagnosis.

Disease features	
Median age (IQR), years	73 (64–76)
Male sex, no. (%)	20 (71.4%)
**Smoking status, no. (%)**	
Non-smoker	6 (23.1%)
Former smoker	9 (34.6%)
Smoker	11 (42.3%)
**Comorbidities, no. (%)**	
HBP	11 (39.3%)
COPD	9 (32.1%)
Dyslipidemia	7 (25.0%)
Atrial fibrillation	4 (16.0%)
Cardiac failure	4 (16.0%)
Diabetes	3 (10.7%)
Psoriasis	2 (7.1%)
OSA	2 (7.1%)
Alcoholism	2 (7.1%)
Asthma	1 (3.6%)
Stroke	1 (3.6%)
Laryngeal carcinoma	1 (3.6%)
Hypothyroidism	1 (3.6%)
GERD	1 (3.6%)
Ankylosing spondylitis	1 (3.6%)
Colon cancer	1 (3.6%)
Organizing pneumonia	1 (3.6%)
Peripheral arterial disease	1 (3.6%)
Scleroderma	1 (3.6%)
Lymphoma	1 (3.6%)
Non-significant	3 (10.7%)
**Histology, no. (%)**	
Adenocarcinoma	13 (46.4%)
Squamous cell carcinoma	7 (25.0%)
Small cell lung cancer	3 (10.7%)
Non–small cell lung carcinoma	1 (3.6%)
Neuroendocrine	1 (3.6%)
Poorly differentiated carcinoma	2 (7.1%)
Glomic tumor	1 (3.6%)
**Staging, no. (%)**	
IV 16/26 (61.5%)	16 (61.5%)
IIIb 4/26 (15.4%)	4 (15.4%)
Ia 1/26 (3.8%)	1 (3.8%)
IIa 1/26 (3.8%)	1 (3.8%)
IIIa 1/26 (3.8%)	1 (3.8%)
IIIb 1/26 (3.6%)	1 (3.6%)
**Median time since LC diagnosis (IQR), months**	6 (1–13)
**Last LC treatment, no. (%)**	
No treatment/recent diagnosis	8 (28.6%)
Carboplatin-vinorelbine	4 (14.3%)
Carboplatin-pemetrexed	3 (10.7%)
Atezolizumab	2 (7.1%)
Docetaxel	2 (7.1%)
Durvalumab	2 (7.1%)
Etoposide	2 (7.1%)
Gencitabine	1 (3.6%)
Carboplatin-Paclitxel	1 (3.6%)
Caboplatin-gemcitabine	1 (3.6%)
Nivolumab	1 (3.6%)
Carboplatin + etoposide	1 (3.6%)
Time since last treatment (days)	16 (13–41)

*IQR, interquartile range; HBP, high blood pressure; COPD, chronic obstructive pulmonary disease; OSA, obstructive sleep apnea; GERD, gastroesophageal reflux; LC, lung cancer.*

Regarding LC histological type, adenocarcinoma was the most common (*n* = 13, 46.4%), followed by squamous cell carcinoma (*n* = 7, 25.0%). Most had stage IV metastatic LC (*n* = 16, 61.5%), whereas four had stage IIIb (15.4%). The remaining histological types and staging are represented in Table 1.

Two patients (7.1%) had a prior history of another cancer, including lymphoma (recently treated with rituximab) and colon cancer. Eleven patients (39.3%) had hypertension, and nine (32.1%) had chronic obstructive pulmonary disease (COPD) (two of them requiring long-term oxygen therapy). Other relevant comorbidities included hypercholesterolemia (*n* = 7), diabetes (*n* = 3), and heart failure (*n* = 3). One patient had organizing pneumonia (OP) undergoing steroid treatment (Table 1).

Most patients were on active treatment (*n* = 20, 71.4%). The anticancer therapy is detailed in Table 1. The median time from the last administration of chemotherapy to COVID-19 diagnosis was of 16 days (IQR = 13–41 days). The patients from the first COVID-19 wave (*n* = 5) had a median time of last treatment to diagnosis of 4 days, whereas the remaining had 18 days (IQR = 13–41 days).

Most patients (11/22) were under corticosteroid therapy, namely, for symptom relief and OP treatment. Three patients (60%) were receiving doses ≥20 mg/day because of OP (*n* = 1), vena cava syndrome (*n* = 1), and for preparing chemotherapy (*n* = 1).

Regarding COVID-19, patients mostly presented with dyspnea (*n* = 12, 42.9%) and cough (*n* = 6, 21.4%). Seven patients (25.0%) were diagnosed as a screening for treatment, whereas two patients were screened because of hospital admissions for other reasons (e.g., uncontrolled pain and hemoptysis). The median time since symptom onset until COVID-19 diagnosis was of 2 days (IQR = 0–4 days). More information regarding symptoms at presentation is detailed in Table 2.

**TABLE 2:** Characterization of symptoms, management, and outcomes of COVID-19 in LC patients.

Characterization of COVID-19	
Median symptoms duration (IQR), days	2 (0–4)
**Symptoms**	
Dyspnea	13 (46.3%)
Cough	9 (32.1%)
Sputum	4 (14.3%)
Anorexia	2 (7.1%)
Odynophagia	1 (3.6%)
Hemoptysis	1 (3.6%)
Prostration	1 (3.6%)
Screening before LC treatment	7 (25.0%)
**Biochemical results, median (IQR)**	
White blood cells, ×10^3^/μL	6.31 (4.2–10.9)
Neutrophils/lymphocytes/eosinophils, ×10^3^/μL	3.9; 0.8; 0.01
Hemoglobin, g/dL	11.4 (1.9)
Platelets, ×10^3^/μL	173 (140–356)
C-reactive protein, mg/L	61 (33–164)
**Treatment, no. (%)**	
Supplementary oxygen	18 (64.3%)
Antibiotics	12 (42.9%)
Steroids	9 (32.1%)
HDQ	4 (14.3%)
HFNC	3 (10.7%)
NIV	1 (3.6%)
CPAP	1 (3.6%)
No treatment	10 (35.7%)
**Outcome, no. (%)**	
Death	7 (25.0%)

*IQR, interquartile range; NIV, non-invasive ventilation; HFNC, high-flow nasal cannula; HDQ, hydroxychloroquine; LC, lung cancer; CPAP, continuous positive airway pressure.*

Laboratory findings on admission are shown in Table 2. Regarding data from blood cells count, neutrophilia was present in 17 (70.8%) of patients, whereas 16 (66.7%) had lymphopenia (80%). All patients had increased levels of C-reactive protein.

**FIGURE 1:**
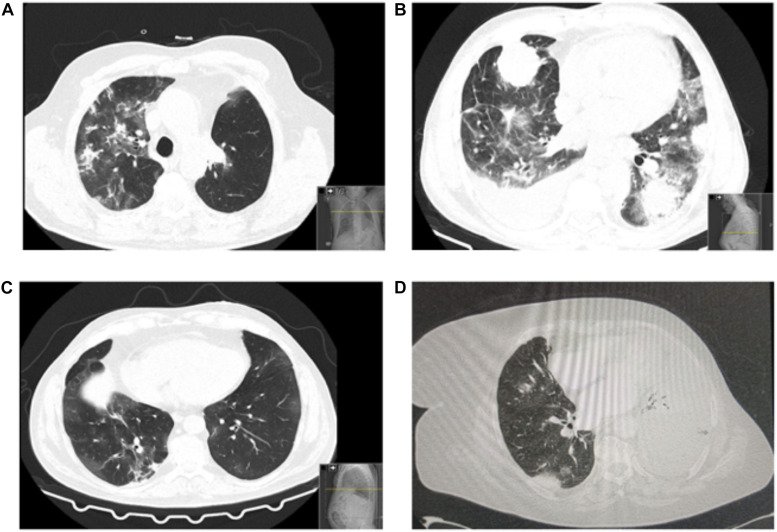
Patients’ chest CT scans. **(A)** Bilateral predominantly peripheral ground-glass opacities. Asymmetrical consolidations in the right lobes suggestive of concomitant bacterial infection. Left upper lobe lobectomy signs (consistent with recent surgery). **(B)** Patient with multiple findings related to coronavirus disease 2019 (COVID-19) and the underlying lung cancer: bilateral ground-glass opacities, pulmonary mass and pleural implant, right pleural effusion. **(C)** Chest CT: bilateral ground-glass opacities, both central and peripheral, some with nodular shape. Some “band” consolidative opacities in the right upper lobe, middle lobe, and lower inferior lobe opacities suggestive of residual processes. **(D)** Left large-volume pleural effusion with left lung atelectasis. Diffuse ground-glass opacities. Small right pleural effusion. No signs of pulmonary thromboembolism.

A total of 16 patients (57.1%) had a thorax image at presentation or shortly after diagnosis of COVID-19. The CT scan of patients revealed the presence of bilateral ground-glass opacities, and three patients evidenced consolidations suggestive of coinfection, in addition to baseline radiological manifestations of LC, such as pleural effusion and lung masses. Figure 1 represents examples of chest CT scan of patients.

Regarding therapeutic intervention, 18 patients (64.3%) received supplemental oxygen, including three (10.7%) who received high-flow nasal cannula at time of intensive care unit admission due to type 1 respiratory failure. One patient (3.6%) needed non-invasive ventilation, and one (3.6%) had continuous positive airway pressure with supplemental oxygen. No patient received mechanical ventilation or extracorporeal membrane oxygenation.

Empirical SARS-CoV-2 treatment with hydroxychloroquine was administered in four cases (14.3%), one of them with concomitant azithromycin. These patients were all treated in the first 3 months. Twelve patients (42.9%) needed empirical antibiotics (two of them in the context of febrile neutropenia). Nine patients (32.1%) were treated with corticosteroids, all in-hospital. One patient remained on corticotherapy at the same dose because of previous OP diagnosis, with CT scan still showing consolidations consistent with the previous diagnosis. This patient developed pulmonary thromboembolism, with no signs of right-sided heart failure and with good recovery after hypocoagulation with low-molecular-weight heparin.

Regarding clinical outcomes, seven patients died (25.0%) after a median time of 5 days since the COVID-19 diagnosis (min–max = 0–116 days). The patients who died had metastatic and progressive LC (stage IV); four of them (80.0%) were previously on steroids.

## Discussion

The COVID-19 pandemic has dramatically changed the population’s daily life and health support, with an increased risk reported for cancer patients. This current scenario has put in a higher emphasis the need for continuous changes and adaptations of previous routines in oncology care units.

In our sample, a high lethality rate was observed. When considering the cases of the first wave (between March and May), in fact, at the end of April, the rate of COVID-19–positive cases for the general population was 0.24%, linked to a lethality rate of 4.0%. However, in our case, the rate per LC patient was 1.12%, where 60% of patients died. Such differences can be attributed, in particular, to the oncological process features, age, treatment regimen, and patients’ comorbidities. For example, in the TERAVOLT study, it was found that, in LC patients, poor prognosis was associated with advanced age (>65 years), comorbidities, Eastern Cooperative Oncology Group performance status grade 2 (ECOG2), steroids (>10 mg of prednisolone), anticoagulants, chemotherapy, and chemotherapy plus immunotherapy (Garassino et al., 2020). In this sample, half of patients were on steroid therapy for different reasons. We could speculate that the worse outcome could also be attributed to the immunosuppression state induced by steroids. Indeed, this aspect further raises the need for a careful intervention in these patients, where the use of lower doses of steroids may be an alternative in cancer patients at high risk of COVID-19.

Additionally, one patient was diagnosed with pulmonary thromboembolism. Cancer is a prothrombotic state that *per se* can explain this finding. However, in COVID-19–derived pneumonia, coagulopathy and disseminated intravascular coagulation are common complications and the most common causes of death (Helms et al., 2020; Wu et al., 2020), with the possible benefit of using heparin in selected acute respiratory distress syndrome patients with coagulopathy (Tang et al., 2020). It is therefore essential to monitor LC patients for the development of pulmonary thrombosis, as they are at an even greater risk of thrombotic events.

In this series, all casualties happened in patients with stage IV LC. This suggests that an advanced and progressive lung disease may be responsible for the worst outcomes. Several reasons may explain the worse prognosis observed to these LC patients, such as the immunosuppression status, male gender, smoking, and the presence of cardiovascular and chronic lung diseases, including the COPD (Cai, 2020; Passaro et al., 2020). LC patients often have recurrent respiratory symptoms, which can eventually delay the recognition of a viral infection for both the patient and the clinician. In our sample, the most common symptoms were dyspnea and cough, which can be wrongly attributed to the disease progression, especially at advanced cancer stages. Thus, regular testing, particularly before treatments and when patients regularly visit the hospital for consultation, should be performed, as they are crucial for early detection, at the same time, which also helps to avoid interventions that can aggravate the disease.

Another key issue is to assess the indication for antineoplastic treatment. In this way, a proper discussion in a multidisciplinary team, which can be done by videoconference and other emerging platforms, can help the clinician to adopt the most appropriate therapeutic option, with a consequent reduction in the risk of infection. In fact, this should be a priority in the decision-making process, if case-by-case risk–benefit assessment is high. In reality, what is intended here is to emphasize that the interruption of the necessary and effective anticancer therapy should not be an option, except for low-priority cases, on a case-by-case analysis. The European Society for Medical Oncology (ESMO) has published specific online guidelines for the different therapeutic regimens that should be considered for LC treatment, dividing them into high (should not be delayed), medium (should not be delayed for more than 6 weeks), and low (the patient’s condition is stable enough that services can be suspended during the pandemic, or the intervention is unlikely to have a marked benefit) priority.

On the other side, and also worth of note is that hospital transmission was considered a possibility in two of the cases, as they underwent treatment at the daycare oncology unit 4 days before the symptoms developed. However, an epidemiologic link has not been found. Other retrospective studies have indicated the occurrence of hospital transmission in COVID-19 patients. For example, in a retrospective case study in China with 138 patients, 41.3% of patients were reported to have acquired SARS-CoV-2 during hospitalization, five in the oncology department (Wang et al., 2020).

In our particular case, since the beginning of the COVID-19 outbreak, and in line with recommendations from international and national societies, such as ESMO and Direção Geral da Saúde, a specific protocol has been designed to be applied to reduce the patients’ access to the oncology unit, promoting telephone screening and consultation. All patients have been screened for COVID-19 symptoms and temperature upon arrival at the entrance to the oncology unit, since the beginning of April. In addition, all patients and staff use a surgical mask inside the hospital. As a result, from May to June 2020, there was no new diagnosis of COVID-19 among LC patients at our institution. This reduction can be in part due to policies adopted to reduce the spread of nosocomial infections. At national level, the peak in the number of active cases was at mid-May (May 15); so, the lack of new cases during the month of May seems to be related to the successful preventive measures implemented. On the other hand, seven patients had a diagnosis as part of screening for oncologic treatment (chemotherapy or radiotherapy), which could have prevented more serious complications had the patients done treatment. This probably explains the findings regarding time, from the last cancer treatment to the COVID-19 diagnosis, having increased from 4 days in the first wave to 16 days in the following months.

As main study limitations, the reduced sample size is the most prominent one, which limits the conclusions regarding clinical presentation and outcomes to the general population of patients with LC. However, this single-center experience majorly intends to highlight the measures related to infection control and its effects on reducing the likelihood of getting infected by SARS-CoV-2 in our patients. Therefore, this analysis adds to the evidence that strict rules to prevent SARS-CoV-2 infection among LC patients are particularly relevant.

## Conclusion

In view of the high vulnerability of LC patients, the death risk associated with COVID-19 is imminent. Thus, and given the data presented in this report, there is urgency in implementing and maintaining protocols at the oncology unit to minimize the viral infection spread and to reduce the consequences in SARS-CoV-2–infected patients.

## Data Availability Statement

The raw data supporting the conclusions of this article will be made available by the authors, without undue reservation.

## Ethics Statement

The studies involving human participants were reviewed and approved by Centro Hospitalar Universitário de São João, Porto, Portugal.

## Author Contributions

All authors listed have made a substantial, direct and intellectual contribution to the work, and approved it for publication.

## Conflict of Interest

The authors declare that the research was conducted in the absence of any commercial or financial relationships that could be construed as a potential conflict of interest.
